# Development of abiotic stress tolerance in inoculated apple plantlets with a plant growth-promoting rhizobacterium, *Bacillus velezensis* strain MWS28 in Korea

**DOI:** 10.3389/fmicb.2026.1842281

**Published:** 2026-07-10

**Authors:** Soh-Young Oh, Kotnala Balaraju, Deok-Hoon Yoon, Chang Hee Lee, Se Weon Lee, Yong Hwan Lee, Kyungseok Park

**Affiliations:** 1 Research Institute of International Agriculture Technology and Information, Hankyong National University, Anseong, Republic of Korea; 2 Plant Disease Control Division, National Institute of Agricultural Sciences, Rural Development Administration, Wanju, Republic of Korea

**Keywords:** abiotic stress tolerance, chilling injury, drought stress, flavonoid biosynthesis genes, PGPR, PR genes

## Abstract

This study investigated the potential of the plant growth-promoting rhizobacterium (PGPR) *Bacillus velezensis* strain MWS28 (MWS28) to enhance abiotic stress tolerance in apple (*Malus domestica*) plantlets in Korea. Treatments with *B. velezensis* MWS28 or *B. vallismortis* EXTN-1 (positive control) by both foliar spraying and dipping methods significantly reduced cold injury symptoms, such as browning and tissue necrosis, compared to untreated controls. MWS28 treatment, especially via foliar spray, suppressed chilling injury by over 85%, outperforming conventional agents like benzothiadiazole (BTH) and streptomycin. Under drought conditions, both MWS28 and EXTN-1 treatments improved relative moisture content (RMC) and minimized leaf wilting throughout the stress period; MWS28-treated plants exhibited the strongest RMC after 15 days of water deprivation. Biochemical analysis showed MWS28-treated plants had significantly elevated activities of antioxidant enzymes (APX, CAT, SOD, and POD) and reduced the malondialdehyde (MDA) levels, indicating enhanced oxidative stress protection and membrane stability. MWS28 treatment also increased endogenous indole-3-acetic acid (IAA) and abscisic acid (ABA) concentrations under stress. Gene expression analysis demonstrated that MWS28 notably upregulated stress-related genes, particularly those involved in antioxidant defense, flavonoid biosynthesis (notably DFR, ANS, and UFGT), and pathogenesis-related (PR5 and PR8) genes in “Fuji” apple leaves using both dipping and foliar spray methods. These results collectively demonstrate that the MWS28 strain confers robust abiotic stress tolerance in apples by enhancing physiological resilience and activating key molecular defenses. This approach offers a promising, sustainable strategy for mitigating climate-related stresses in apple cultivation.

## Introduction

Abiotic stresses, including drought, salinity, extreme temperatures, and heavy metal toxicity post substantial challenges to several crops including apple (*Malus domestica*) on a global scale (Li and Ahammed, 2023). These challenges are particularly acute in Korea, where climate change has led to unstable weather patterns that disrupt apple cultivation and intensify the impacts of abiotic stresses (Etesami et al., 2023). Among these, cold and drought are the most limiting factors, with cold stress during sensitive stages, especially flowering, can cause severe flower and fruit damage, tissue browning, necrosis, and reduced yield and quality (Hussain et al., 2018; Zhou L. et al., 2025). Drought stress further impairs physiological processes, leading to reduced plant vigor, leaf wilting, and decreased productivity (Farooq et al., 2009), while prolonged drought can reduce water uptake, disrupt photosynthesis, alter flowering, lower fruit quality, and even result in tree death. The combined effect of these stresses, exacerbated by climate change, leads to decreased apple yields and compromised fruit quality (Etesami et al., 2023).

Recent studies have shown that losses in major crops due to abiotic stressors have increased sharply, posing serious risks to food security (Bibi and Rahman, 2023; Begna and Wakweya, 2025). The severity of plant responses to these stresses is influenced by the duration, intensity, and the growth stage of the plant (Feller and Vaseva, 2014). Cold stress, in particular, is a major factor limiting the growth and development, thereby threatening global food security (Roychowdhury et al., 2025; Qian et al., 2024). Cold stress disrupts crop production by causing physiological and metabolic disturbances such as ROS accumulation, nutritional imbalances, membrane damage, reduced photosynthesis, and hormonal imbalance (Egamberdieva et al., 2017; Guy et al., 2008). In response, plants activate a variety of stress-related genes, enhancing freezing tolerance and regulating gene expression and signal transduction under cold conditions (Yamaguchi-Shinozaki and Shinozaki, 2006; Kidokoro et al., 2017).

In recent years, the apple industry in South Korea has experienced substantial losses due to cold damage, with unexpected spring frosts posing a major threat; between 2018 and 2023, frost events accounted for 63% of all fruit crop insurance claims, and apple production declined by over 30% in 2023 (Choi et al., 2025). Major apple-producing regions, such as Gyeongnam experienced severe blossom and fruit set damage following sudden temperature drops after early blooming, a phenomenon worsened by climate change and phenological shifts (Lee et al., 2023). Rising temperatures have led to earlier blooming, which increases vulnerability to late frosts, particularly in mid-mountainous regions (Choi et al., 2025). Consequently, national apple production declined sharply from 566,000 tons in 2022 to 394,000 tons in 2023, resulting in higher prices and economic difficulties for producers (KED Global, 2024). These challenges highlight the urgent need for adaptive measures, such as smart orchard technologies and development of frost-tolerant cultivars (Zhou et al., 2021; Choi et al., 2025).

Drought stress causes plants to accumulate reactive oxygen species (ROS), necessitating strong molecular defenses; antioxidant genes encoding enzymes such as superoxide dismutase (SOD), catalase (CAT), and ascorbate peroxidase (APX) detoxify ROS (Gill and Tuteja, 2010; Mittler et al., 2004), while flavonoid biosynthesis genes such as chalcone synthase (CHS), chalcone isomerase (CHI), and flavonol synthase (FLS) help scavenge ROS, and pathogenesis-related (PR) genes stabilize membranes and support stress signaling during drought (Agati et al., 2012; Nakabayashi et al., 2014). The coordinated expression of these genes is essential for effective drought resistance.

To address the challenges posed by abiotic stress, plant growth-promoting rhizobacteria (PGPR), such as *Bacillus velezensis* and *Bacillus vallismortis* have been shown to induce stress tolerance in plants (Vurukonda et al., 2016; Park et al., 2024; Hashem et al., 2019). These bacteria help plants adapt to environmental stresses by regulating stress-related genes, enhancing antioxidant activity, and modulating plant hormones (Etesami et al., 2023). While PGPR have demonstrated effectiveness in developing cold and drought tolerance in crops such as wheat (Kasim et al., 2013), maize (Sandhya et al., 2010), and rice (Mahreen et al., 2023), their potential in developing such tolerances in apple flowers and plants remains largely unexplored. Building on these insights, the present study investigates the use of PGPR strain to enhance abiotic stress tolerance in apples, with the goal of establishing a sustainable and eco-friendly cultivation strategy.

## Materials and methods

### Bacterial strains

Bacterial strains used in this study were *Bacillus velezensis* MWS28 and *Bacillus vallismortis* EXTN-1 (positive control), both isolated from different soil rhizospheres. MWS28 was isolated from grapevine rhizosphere soil following a method described by Jin et al. (2018). In brief, soil samples were collected from the root zones of the healthy grapevines at Hankyung University. To enhance the recovery of endospore-forming *Bacillus* species, 1 g of soil was mixed in 9 mL of sterile distilled water (SDW) and incubated at 80°C for 60 min. This heat treatment is to inhibit the non-spore-forming bacteria, especially Gram-negative bacteria. After heat treatment, serial dilutions (10^–2^ to 10^–4^) were prepared. Aliquots (1 mL) were plated onto tryptic soy agar (TSA) plates and incubated at 28°C for 48 h. Distinct colonies were selected, and purified by streaking onto fresh medium, and preserved in tryptic soy broth (TSB) containing 20% glycerol at −80°C. Out of 230 isolates screened for antagonistic activity against the grape white rot pathogen, the strain MWS28 (accession no. MK208682), exhibited significant inhibitory effects and was identified by 16S rRNA gene sequencing. EXTN-1 was isolated from red-pepper rhizosphere soil following the method described by Park et al. (2001).

### Cold damage inhibition effect test

When the apple trees were in full bloom, the flowering branches were cut from the main branch and brought to the laboratory under refrigeration conditions. The branches were treated with bacterial suspensions by dipping or foliar spray method. The branches were selected based on the presence of flowers at a uniform level, and five branches were used per treatment. To prepare bacterial suspensions, cultures from –80°C were grown on TSA plates at 28°C for 24 h. Single colonies were then transferred to a 50-mL Falcon tubes containing 30 mL of TSB. The Falcon tubes were incubated at 28°C for 24 h under shaking conditions (180 rpm). The tubes were then centrifuged at 10,000 × *g*, and the pellet was dissolved in SDW to adjust to a concentration of 10^6^ CFU/mL. In the dipping method, apple stem cuttings bearing flowers were immersed in Falcon tubes containing bacterial suspensions of MWS28 or EXTN-1 (positive control) at a concentration of 10^6^ CFU/mL, as verified by plating and colony enumeration. The cuttings were submerged for 1 min. For the spray treatment; a suspension of the same concentration was applied to apple stem cuttings by spraying, and subsequently stored at room temperature for 24 h, and then kept at –2°C for 2 h. The incidence of cold injury was evaluated 48 h after incubation at room temperature. Non-inoculated control plantlets were dipped/sprayed in SDW. The extent of ovary browning was assessed using 10 flowers per treatment, and each experiment was conducted in triplicate. The experiment was conducted using a randomized block design.

### Chilling injury tolerance test in bacteria-treated stem cuttings

A low-temperature treatment was administered 24 h following the bacterial treatment. Stem cuttings with flowers were kept at –1.5°C for 1 h and 30 min or at –2°C for 2 h. After low-temperature treatment, the stem cuttings were incubated at room temperature for 48 h. The investigation was conducted according to the agricultural science and technology research and analysis standards of the Rural Development Administration. The crown of flowers was cut vertically with a sharp knife and observed with a magnifying glass (10X), and the discoloration of the ovary to brown was evaluated as cold damage. The results were compared with both the non-treated control (water) and the positive control (EXTN-1). The experiment was conducted using a randomized block design.

### Effect of bacterial treatment on drought stress tolerance

To investigate the effect of bacterial treatment on drought stress tolerance, relative moisture content (RMC) was determined following the protocol by Jin et al. (2018) using 3-year-old apple plants (cv. Fuji) grown in pots containing unmodified orchard soil. Plants were divided into two groups: a control group, which received 1 L of tap water every 3 days, and a treatment group, which received 1 L of a diluted suspension (10^6^ CFU/mL) of bacterial strains MWS28 or EXTN-1 (positive control) at the same interval. After 15 days of irrigation, watering was discontinued to induce drought stress. Growth, saturated weight, and dry weight were measured at 3-day intervals post-irrigation cessation. Leaf samples were collected from the 3rd and 4th positions around the shoot apex between 3:00 and 4:00 pm; 10 mm discs centered on the leaf vein were excised from each leaf. Fresh weight was measured immediately after sampling. To determine saturated weight, samples were placed in a 50 mL Falcon tube containing 40 mL of distilled water and incubated at room temperature for 24 h. The dry weight was measured after drying the sample at 70°C for 48 h. Water content was assessed from leaves of three plants per treatment, and mean values were reported. The experiment was conducted using a randomized block design.

### Determination of antioxidant enzyme activities

After 48 h of bacterial treatment, both bacteria-treated and untreated apple stem cuttings were exposed to chilling stress at –2°C for 2 h. Leaf samples were collected 48 h after incubation. The collected leaves were washed gently with distilled water and blot-dried. For the extraction of antioxidant enzymes such as ascorbate peroxidase (APX), catalase (CAT), superoxide dismutase (SOD), and peroxidase (POD), about 0.5 g of fresh leaf tissue was homogenized in a mortar and pestle with 5 mL ice-cold extraction buffer containing 50 mM potassium phosphate buffer (pH 7.0) and 0.4% (w/v) polyvinylpyrrolidone (PVP). The homogenate was filtered through cheesecloth and centrifuged at 10,000 × *g* for 10 min at 4°C. The resulting supernatant was collected and used as the crude enzyme extract for the determination of APX, CAT, SOD, and POD activities using a UV/VIS spectrophotometer (Shimadzu UV-1900i, Japan). All extraction procedure was carried out at 4°C to minimize enzyme degradation.

APX and CAT activities were determined following the method described by Batool et al. (2020). For APX activity, 200 μL of crude enzyme extract was added to a reaction mixture containing 50 mM potassium phosphate buffer (pH 7.0), 0.5 mM ascorbic acid (ASC), and 0.1 mM H_2_O_2_. The decrease in absorbance at 290 nm, resulting from the oxidation of ascorbate, was monitored for 1 min using a UV–Visible spectrophotometer. APX activity was calculated based on the rate of ascorbate oxidation. The CAT activity was assayed by mixing 200 μL enzyme extract with a reaction mixture consisting of 1.5 mL of 50 mM sodium phosphate buffer (pH 7.8), 300 μL of 0.1 M H_2_O_2_, and 1.0 mL of distilled water. The decomposition of H_2_O_2_ was measured by recording the decrease in absorbance at 240 nm per minute, and the CAT activity was expressed accordingly. The SOD activity was determined according to the method of Tang et al. (2010). The reaction mixture contained 0.1 mL enzyme extract, 1.5 mL of 50 mM sodium phosphate buffer (pH 7.8), 0.3 mL of 130 μM methionine, 0.3 mL of 750 μM nitro blue tetrazolium (NBT), 0.3 mL of 100 μM EDTA-Na_2_, 0.3 mL of 20 μM riboflavin, and 100 μL of distilled water. The reaction mixture was illuminated under fluorescent light (4,000 lux) for 20 min, and absorbance was recorded at 560 nm. One unit of SOD activity was defined as the amount of enzyme required to inhibit 50% of NBT. The POD activity was determined by adding 50 μL of enzyme extract to a reaction mixture containing 1.0 mL 50 mM sodium phosphate buffer (pH 5.5), 1.0 mL 0.3% H_2_O_2_, and 0.95 mL of 0.2% guaiacol. The increase in absorbance due to the formation of tetraguaiacol was monitored at 470 nm, and POD activity was calculated from the change in absorbance per minute.

### Lipid peroxidation

Lipid peroxidation was estimated by determining the malondialdehyde (MDA) content using the thiobarbituric acid (TBA) reaction following the method described by Hernández and Almansa (2002). After 48 h of bacterial treatment and water-treated control, approximately 200 mg of fresh leaf tissue was homogenized in 2 mL of 0.1% (w/v) trichloroacetic acid (TCA). The homogenate was centrifuged at 13,000 × *g* for 20 min at 4°C using a refrigerated centrifuge (HERMLE Z 36 HK). Subsequently, 0.5 mL of the resulting supernatant was mixed with 1.5 mL of 0.5% (w/v) TBA prepared in 20% (w/v) TCA. The reaction mixture was incubated at 95°C for 30 min and then immediately cooled in an ice-water bath to terminate the reaction. The samples were centrifuged at 10,000 × *g* for 10 min, and the absorbance of the clear supernatant was read at 532 and 600 nm using a UV-Visible spectrophotometer.

Lipid peroxidation concentration was determined as follows:

MDA μmoL g^–1^ = [(OD 532–OD 600) × TV)/(e × DW)].
TV = Total volume of the extract (2 mL);
e = 155 mM^–1^ cm^–1^; DW = Dry weight (g).
Dry weight (DW) = Fresh weight (FW)–[(WC/100) × Fresh weight (FW)].

### Determination of indole-3-acetic acid (IAA) and abscicic acid (ABA)

The contents of IAA and ABA were determined from fresh leaf tissues following methanolic extraction. Approximately 0.5 g of fresh plant tissue collected from rhizobacteria-treated plants was homogenized in 5 mL of cold 80% (v/v) methanol and incubated at 4°C for 12 h. The homogenate was centrifuged at 10,000 × g for 10 min, and the resulting supernatant was collected for hormone analysis.

For IAA determination, the colorimetric method developed by Glickmann and Dessaux (1995) was employed. Briefly, 1 mL of the methanol extract was mixed with 2 mL of Salkowski reagent prepared from 1 mL of 0.5 M FeCl_3_ dissolved in 50 mL of 35% perchloric acid. The reaction mixture was incubated at room temperature for 30 min in the dark. Development of a pink coloration indicated the presence of IAA, and absorbance was recorded at 530 nm using a UV–Visible spectrophotometer. The IAA concentration was calculated from a standard curve prepared using known concentrations of authentic IAA standards. For ABA determination, 1 mL of methanolic extract was mixed with 2 mL of ferric chloride reagent containing 0.5% FeCl_3_ in 0.1 N HCl. After incubation at room temperature for 30 min, the absorbance was measured at 410 nm using a UV–Visible spectrophotometer. ABA concentration was estimated using a calibration curve generated from standard ABA solutions. All measurements were performed in triplicate, and the results were expressed as μg g^−1^ fresh weight (FW).

### Total RNA extraction and gene expression analysis

For total RNA extraction and gene expression analysis, apple stem cuttings were treated either by soaking or spraying with suspensions of MWS28 or EXTN-1 strains, prepared from 24-h-old cultures. Twenty 4 h after treatment with bacterial suspensions, leaf samples were collected, immediately frozen in liquid nitrogen, and stored at –72°C until further use. Total RNA was isolated using Ribospin Seed/Fruit Kit ver 1.1 (GeneAll, Korea), according to manufacturer’s instructions. Total RNA concentration (μg/μL) and purity (A260/A230 and A260/A280 ratio) were determined using a Nanodrop 2000 spectrophotometer (Thermo Fisher Scientific, United States). Gene expression was analyzed by quantitative real-time PCR (qRT-PCR) in three biological replicates. Primers targeting antioxidant-related genes (AKR, APX, and CAT), flavonoid biosynthesis pathway-related genes (PAL, CHS, CHI, F3H, F3’H, FLS, DFR, LAR, ANR, ANS, and UFGT), and pathogenesis-related genes (PR1, PR2, PR5, and PR8) were designed based on the study of Park et al. (2023). The list of primers was provided in Table 1. The cDNA was synthesized from total RNA using a QuantiTect Reverse Transcription Kit (QIAGEN, Germany) following the manufacturer’s instructions. The qRT-PCR was performed using the QuantStudio 3 system (Thermo Fisher Scientific, United States), under the following conditions: initial denaturation at 95°C for 2 min, followed by 40 cycles of 95°C for 10 s, 60°C for 15 s, and 72°C for 20 s. Gene expression levels were analyzed in triplicate using the relative quantification method (2^–ΔΔCt^; Livak and Schmittgen, 2001), with Actin (Accession Number: XM_008356922.3) serving as the internal control.

**TABLE 1:** List of primers used in this study.

Gene	Accession no.	Sequence (5′–3′)
PAL	XM_008389362.3	F: AGGAACACCGTAAAGAACA R: ACATACTCCCTATCGACAAC
CHS	XM_029091251.1	F: GGCACAAACCATTCTCGCTG R: TGAAGGCCTCGTTAAGGCTC
CHI	XM_017330968.2	F: GTCAAGGAATTACCGACATTGA R: CACAACTTCAGGGTCCAAGTAA
F3H	NM_001293925.1	F: TGGAAGGAGCTTTTGTGGTCA R: CTGGGTTCTGGAATGTGGCT
F3’H	XM_008376388.3	F: TGCCACTCTTCTGGTCAACG R: TCATTACCCTTGACGTCCACA
FLS	NM_001319250.1	F: GGAGTCTGTGGAGAGAGAACG R: CCAAGACTTTCCCATGAACGG
DFR	NM_001293939.1	F: TCGACGACAACTTAGAACCAG R: AATCGGAATCGGAATCAAGCC
LAR	NM_001294114.1	F: CTTGGATGAGTGCTTCGATGG R: TGACAGTCCTGGATTCGACAA
ANR	XM_029110431.1	F: CATCTGGTCGGTACATTTGCTG R: ATGATCAGTTTGGCCTCAGAC
ANS	NM_001293838.1	F: CTCCTTTGGTGGCTCACAGA R: CATGCACATTGGGGACACAC
UFGT	NM_001293991.1	F: ACCTTCCAACGTAGCATCCC R: GCCATTGTCCCGAAGCTGAT
AKR	XM_008382208.2	F: AGGAGGATCAAGTTGGGTTCTCA R: GTGGAGAGCGATCATGTCTTCAT
APX	XM_008387034.2	F: CCTGATGCTACCAAGGGTTGTG R: TCCAGATCGCTCCTTGTGGC
CAT	XM_008350702.2	F: GCTCGAAGGAAGTCAGCACA R: CTGGTGGAGAAACTCGCCAA
PR1	XM_008372355.3	F: GCAGCAGTAGGCGTTGGTCCCT R: CCAGTGCTCATGGCAAGGTTTT
PR2	XM_008368189.3	F: CACTGACCCTGCAAACCAAT R: AGGCAAGGTCTATGCTACCAG
PR5	XM_029108558.1	F: AACTAGCATCCAAAGCTAGCC R: CCACAGTCTGCAGTTTCACAAG
PR8	XM_008364574.3	F: ACTTGCCTTGCATCATCAGC R: CCCTAGTGGTACTTGCACGG

F, forward; R, reverse. Primer sequences from Park et al. (2023).

### Statistical analysis

The experimental results were analyzed for significance using two-way ANOVA in SAS Enterprise Guide 9.4 (SAS Institute, Inc., Cary, United States. Significant differences between treatment means were determined using the least significant difference (LSD) test at *P* ≤ 0.05. All experiments were performed at least twice.

## Results

### Cold damage suppression effect by bacterial treatment

**FIGURE 1:**
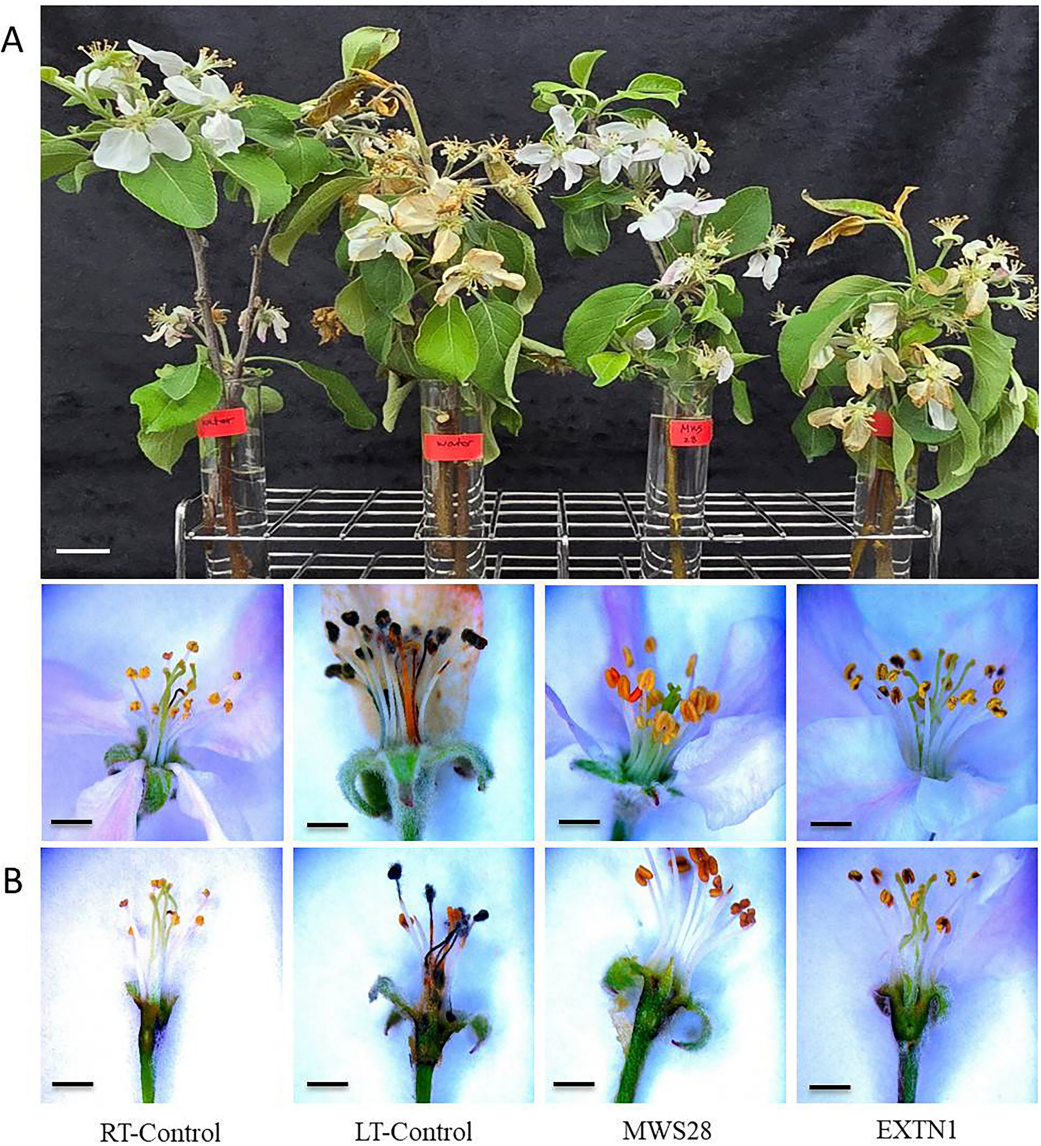
Effect of bacterial treatment on apple flowers against cold damage. Apples flowers were treated with selective bacteria *B. velezensis* MWS28 and *B. vallismortis* EXTN-1 (positive control) by foliar spray (*Bar* 1.0 cm) **(A)**, and dipping **(B)** methods at the concentration of 10^6^ CFU/mL. Stem cuttings with treated flowers were kept at room temperature for 24 h, then stored at -2°C for 2 h, and examined for cold injury symptoms after 48 h at room temperature, and compared with a non-treated control. RT-Control: flowers kept at room temperature; LT-Control: flowers treated with water and exposed to cold; MWS28: flowers treated with *B. velezensis* MWS28; EXTN1: flowers treated *B. vallismortis* EXNT-1 (positive control). Flowers at room temperature remained healthy, while chilling injury was observed in water-treated controls (*Bar* 1.0 cm).

The effects of bacterial treatments on cold damage in apple plantlets were evaluated using *Bacillus velezensis* MWS28 and *B. vallismortis* EXTN-1. Application of both bacterial strains, either by foliar spray or dipping, effectively reduced the cold injury symptoms compared with the untreated controls. Flowers maintained at room temperature (RT-Control) remained healthy and showed no visible damage, whereas water-treated flowers exposed to low temperature (LT-Control) exhibited severe browning and tissue necrosis. On the other hand, flowers treated with MWS28 or EXTN-1 prior to cold exposure exhibited relatively less browning and retained a healthier appearance, indicating enhanced tolerance to cold stress (Figure 1).

**FIGURE 2:**
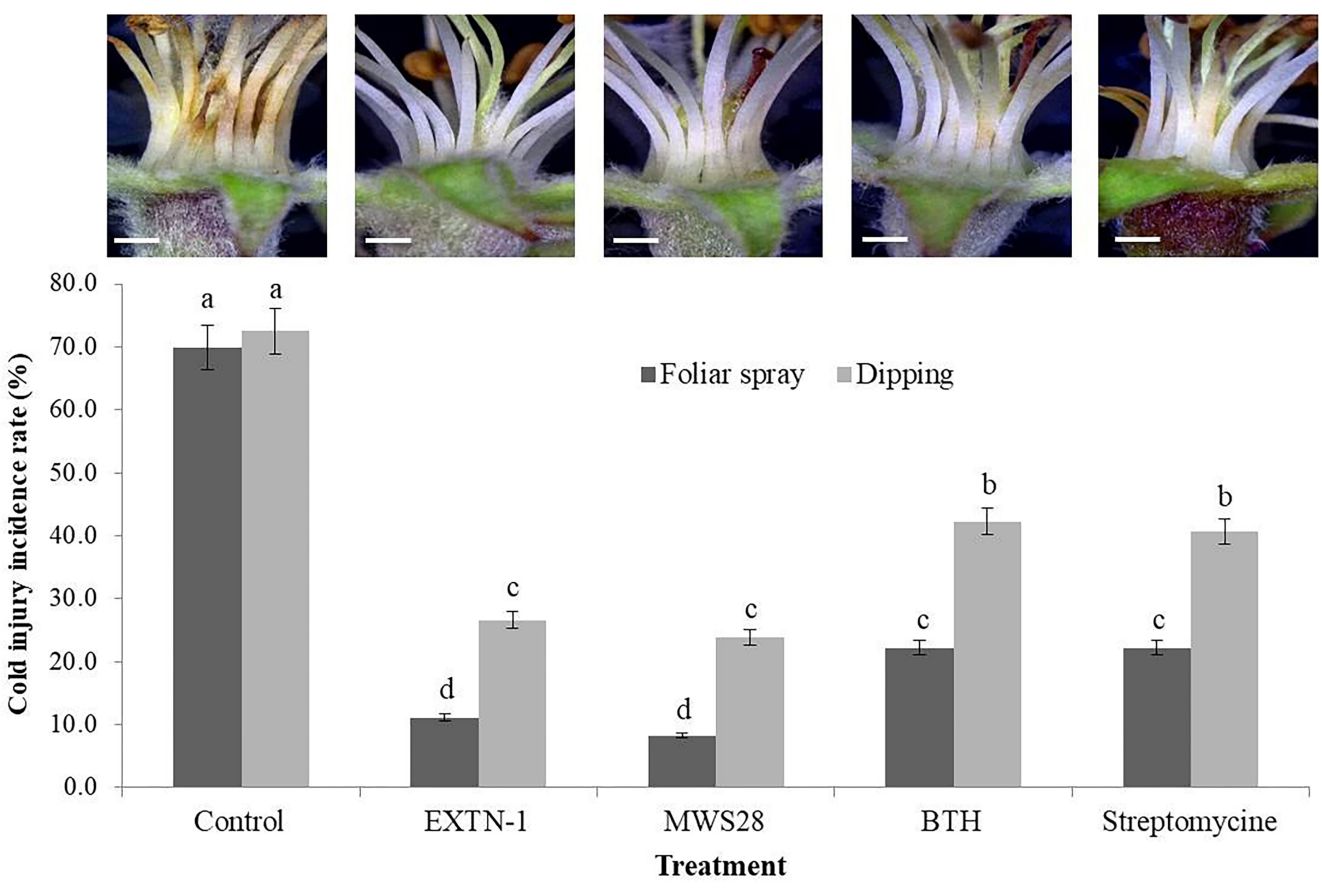
Development of tolerance to chilling injury in apple flowers following bacterial treatment. Chilling injury incidence (%) in apple flowers treated with *B. velezensis* MWS28 or *B. vallismortis* EXTN-1 (positive control) by foliar spray and dipping methods and exposed to -1.5°C for 1 h and 30 min. Flowers were evaluated 24 h after incubation at room temperature. Treatments included non-treated controls, EXTN-1 (positive control), MWS28, BTH, and streptomycin. Bars represent mean ± SE (*n* = 10). Different letters above bars indicate statistically significant differences (LSD, *P* < 0.05). Flower images were shown only for foliar spray treatment (*Bar* 1.0 cm).

The development chilling tolerance was further confirmed by the reduced incidence of chilling injury in all bacterial treatment groups compared with the untreated control (Figure 2). Foliar spray application exhibited protection against chilling injury relatively a little more than dipping method. The untreated control exhibited the highest incidence of chilling injury, whereas MWS28 and EXTN-1 treatments resulted in the lowest levels of damage. Although BTH and streptomycin treatments also reduced chilling injury, their effectiveness was lower than that of the bacterial treatments. Among all treatments, foliar application of MWS28 showed the strongest suppression of chilling injury, followed closely by EXTN-1. Approximately, 70% of flowers in the untreated control exhibited browning symptoms, whereas incidence was reduced to 8 and 10% in flowers treated with MWS28 and EXTN-1, respectively. These reductions correspond to more than 85% suppression of chilling injury relative to the untreated control. The representative flower images presented in Figure 2 corroborate these findings, showing substantially healthier floral tissues in the MWS28- and EXTN-1-treated groups than in the control. These findings indicate that bacterial treatments effectively reduced cold-induced damage in apple flowers, with MWS28 providing the highest level of protection against chilling injury.

### Effect of bacterial treatment on drought stress tolerance in apple plants

**FIGURE 3:**
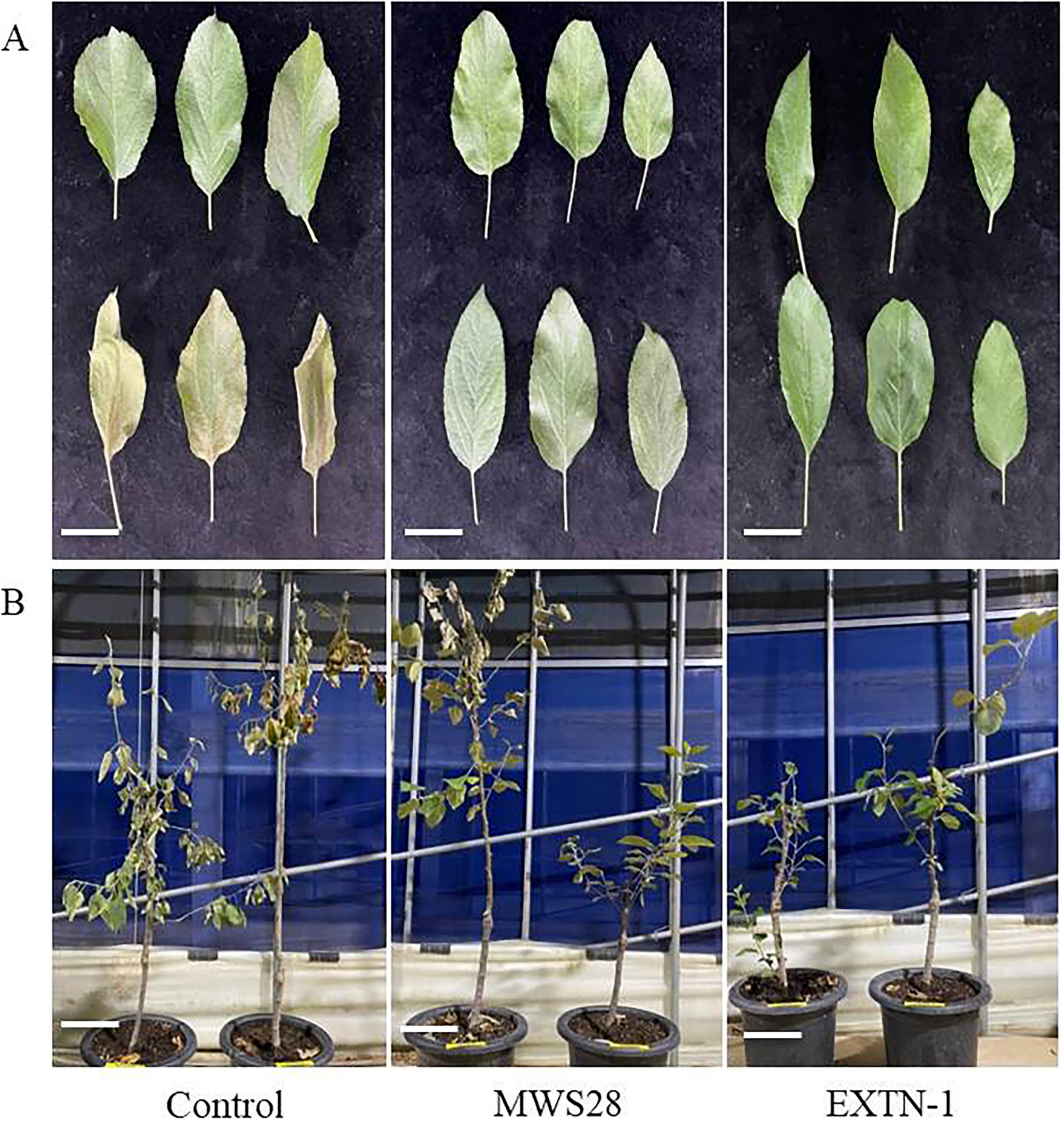
Effect of bacterial treatment on drought stress tolerance in apple plants. **(A)** Determination of leaf wilting after 15 days of irrigation, following treatment with bacterial suspensions of MWS28 and EXTN-1 (positive control). Representative leaves from the control, MWS28, and EXTN-1 groups illustrate differences in wilting symptoms (*Bar* 2.0 cm). **(B)** Comparison of plant recovery 3 days after rewatering. The overall conditions of plants treated with MWS28 and EXTN-1 is visually compared with untreated controls. All treatments were compared with a non-treated control group (*Bar* 5.0 cm).

Bacterial treatments significantly improved drought tolerance in apple plants compared to the untreated control (Figure 3). After 15 days without irrigation, leaves of the untreated control plants exhibited severe yellowing and wilting, whereas plants treated with MWS28 or EXTN-1 showed considerably less wilting and maintained a healthier appearance (Figure 3A). Following rewatering, bacterial-treated plants recovered more rapidly and exhibited greater vigor than untreated plants (Figure 3B). Differences in drought tolerance were further reflected in the relative moisture content (RMC) of the leaves (Table 2). In the untreated control, RMC declined progressively from 56.46% on day 3 to 29.79% on day 15 after irrigation was stopped. In contrast, plants treated with MWS28 and EXTN-1 maintained significantly higher RMC values throughout the stress period. The effect became particularly evident after 12 days of drought stress, when MWS28- and EXTN-1-treated plants retained the highest RMC (53.26%), followed by EXTN-1-treated plants (47.12%), whereas the control plants showed substantial decline to 29.79%. Overall, bacterial treatments mitigated the effects of drought stress by reducing leaf wilting, maintaining higher leaf moisture content, and promoting faster recovery after rewatering. Among the treatments, MWS28 consistently provided the greatest improvement in drought tolerance.

**TABLE 2:** Comparison of changes in relative moisture content (RMC) of apple leaves after irrigation suspension.

Treatment	Relative moisture content (%)^y^				
	3^rd^ day	6^th^ day	9^th^ day	12^th^ day	15^th^ day
Water control	56.46 ± 2.36^a^	54.09 ± 2.91^a^	54.67 ± 1.69^a^	36.64 ± 1.72^a^	29.79 ± 2.40^a^
MWS28	52.64 ± 0.89^a^	54.09 ± 1.11^a^	50.82 ± 2.98^a^	51.57 ± 1.42^b^	53.26 ± 4.72^b^
EXTN-1	55.81 ± 4.56^a^	55.50 ± 1.83^a^	51.17 ± 4.23^a^	51.42 ± 2.27^b^	47.12 ± 1.55^b^

^y^Values are expressed as mean ± SD (standard deviation), which indicates the variability among the replicates for each treatment. Different letters within the columns indicate statistical differences at the 5% levels by Duncan’s multiple range test (DMRT).

### Effect of *Bacillus velezensis* MWS28 treatment on antioxidant enzyme activities under chilling stress

**FIGURE 4:**
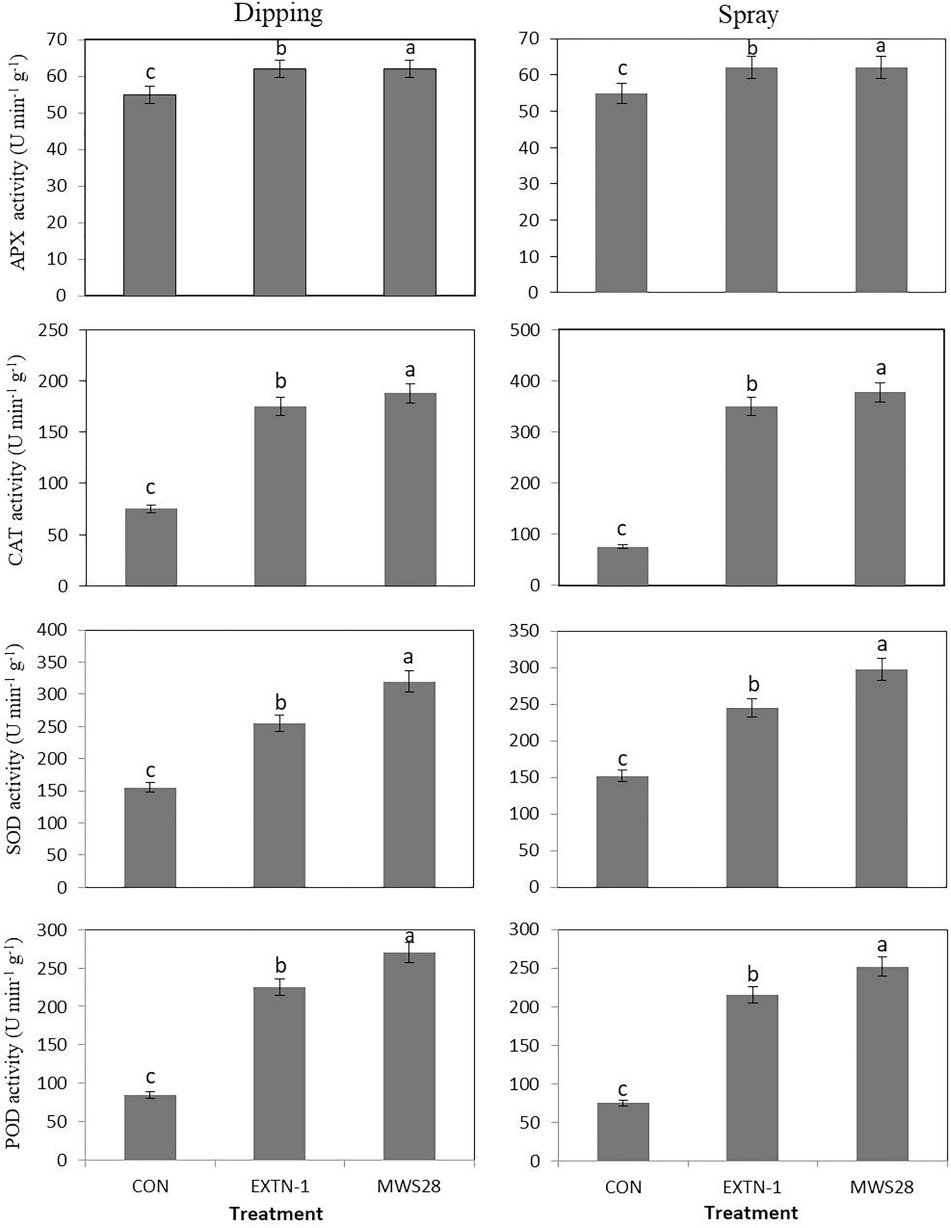
Effect of chilling stress and PGPR applications on enzymatic activities of APX, CAT, SOD and POD in apple stem cuttings under dipping or spray methods. After 48 h of bacterial treatment, both bacteria-treated and untreated apple stem cuttings were exposed to chilling stress at –2°C for 2 h, and leaf samples were collected 48 h after incubation. The crude enzyme extracted the leaf sample was used for the determination of the above enzymes. Enzymatic activities of MWS28-treated samples were compared with non-treated control and positive control (EXTN-1). Values represent the mean ± SE of five biological replicates (*n* = 5). Different letters above bars indicate significant differences among treatments according to the least significant difference (LSD) test at *P* ≤ 0.05.

**FIGURE 5:**
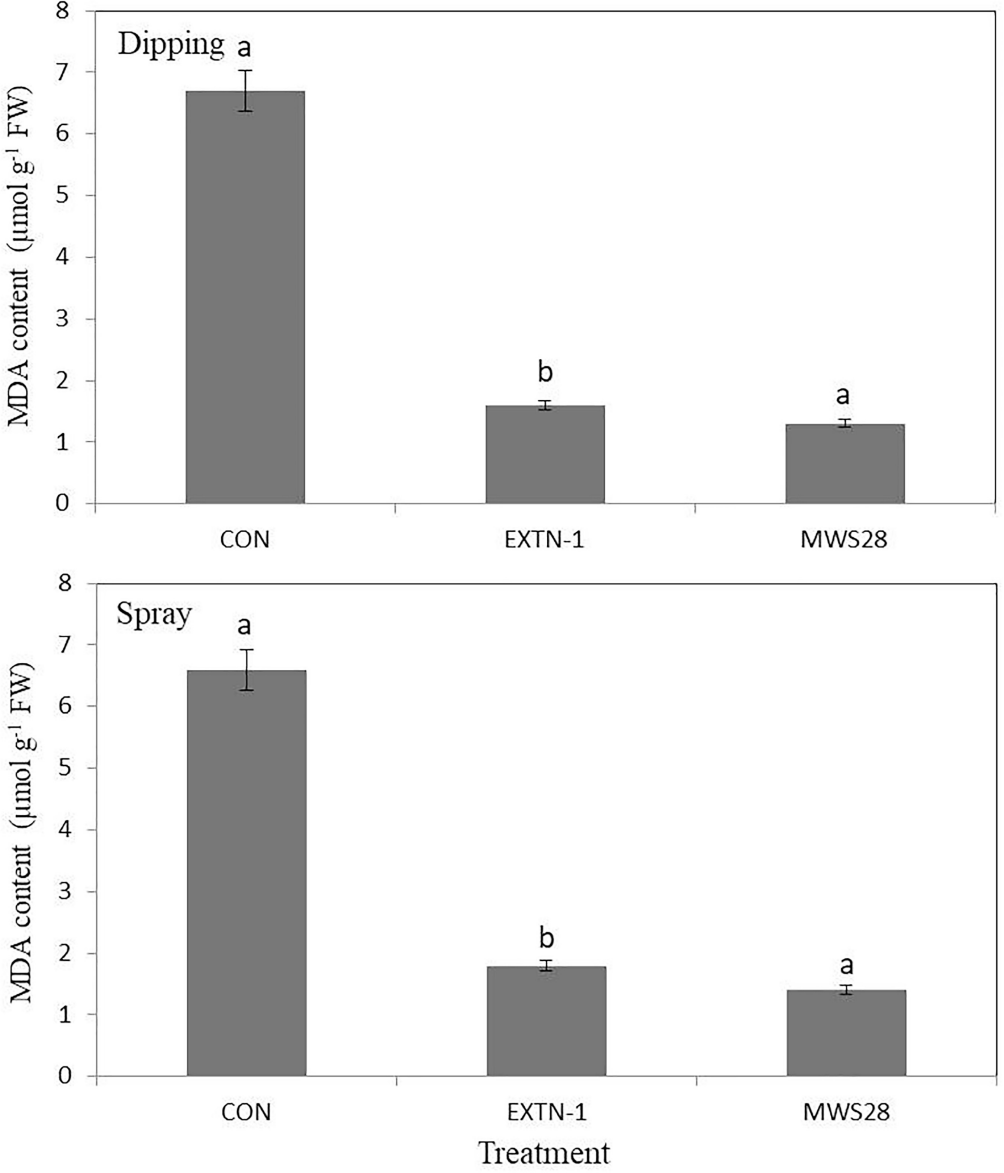
Effect of *Bacillus velezensis* MWS28 application on malondialdehyde (MDA) content in apple stem cuttings subjected to chilling stress. Bacterial treatments were applied through stem dipping and foliar spray methods and compared with the non-treated control (CON) and the positive control strain EXTN-1. MDA content was measured as an indicator of lipid peroxidation and oxidative membrane damage under chilling conditions. Values represent the mean ± SE of five biological replicates (*n* = 5). Different letters above bars indicate significant differences among treatments according to the least significant difference (LSD) test at *P* ≤ 0.05.

Chilling stress markedly influenced the antioxidant defense system of apple stem cuttings, and bacterial treatments significantly (*P* ≤ 0.05) enhanced the activities of antioxidant enzymes (Figure 4). In both stem-dipping and foliar-spray applications, plants treated with MWS28 exhibited the highest activities of ascorbate peroxidase (APX), catalase (CAT), superoxide dismutase (SOD), and peroxidase (POD), followed by the positive control strain EXTN-1, whereas untreated control plants showed the lowest activities. APX activity increased substantially in MWS28-treated plants, reaching approximately 55–60 U min^−1^ g^−1^ compared with 25–35 U min^−1^ g^−1^ in the untreated control. Similarly, CAT activity was significantly (*P* ≤ 0.05) higher in MWS28-treated plants than in both EXTN-1-treated and untreated plants. SOD activity showed the most pronounced increase, particularly under foliar spray treatment, where MWS28-treated plants exhibited nearly a two-fold increase relative to the control. POD activity also followed a similar trend, with significantly higher enzyme activity in MWS28-treated plants than in both EXTN-1-treated and untreated plants (Figure 5). MWS28 consistently induced the highest antioxidant enzyme activities among all treatments, indicating enhanced enzymatic defense against chilling-induced oxidative stress.

### Effect of MWS28 treatment on lipid peroxidation content under chilling stress

The accumulation of malondialdehyde (MDA), an indicator of membrane lipid peroxidation, was significantly affected by bacterial treatments under chilling stress (Figure 5). In both dipping and spray application methods, untreated control plants exhibited the highest MDA content, reaching approximately 6.5–7.0 μmoL g^−1^ FW. Application of the positive control strain EXTN-1 significantly reduced MDA accumulation compared with the untreated control. The lowest MDA levels were observed in MWS28-treated plants, with values reduced to approximately 2.0–3.0 μmoL g^−1^ FW depending on the application method. Foliar spray treatment with MWS28 resulted in a slightly greater reduction in MDA content than stem dipping. Significant differences were detected among all treatments according to the LSD test (*P* ≤ 0.05), indicating that MWS28 effectively suppressed chilling-induced oxidative membrane damage.

### Effect of MWS28 treatment on endogenous IAA and ABA contents under chilling stress

**FIGURE 6:**
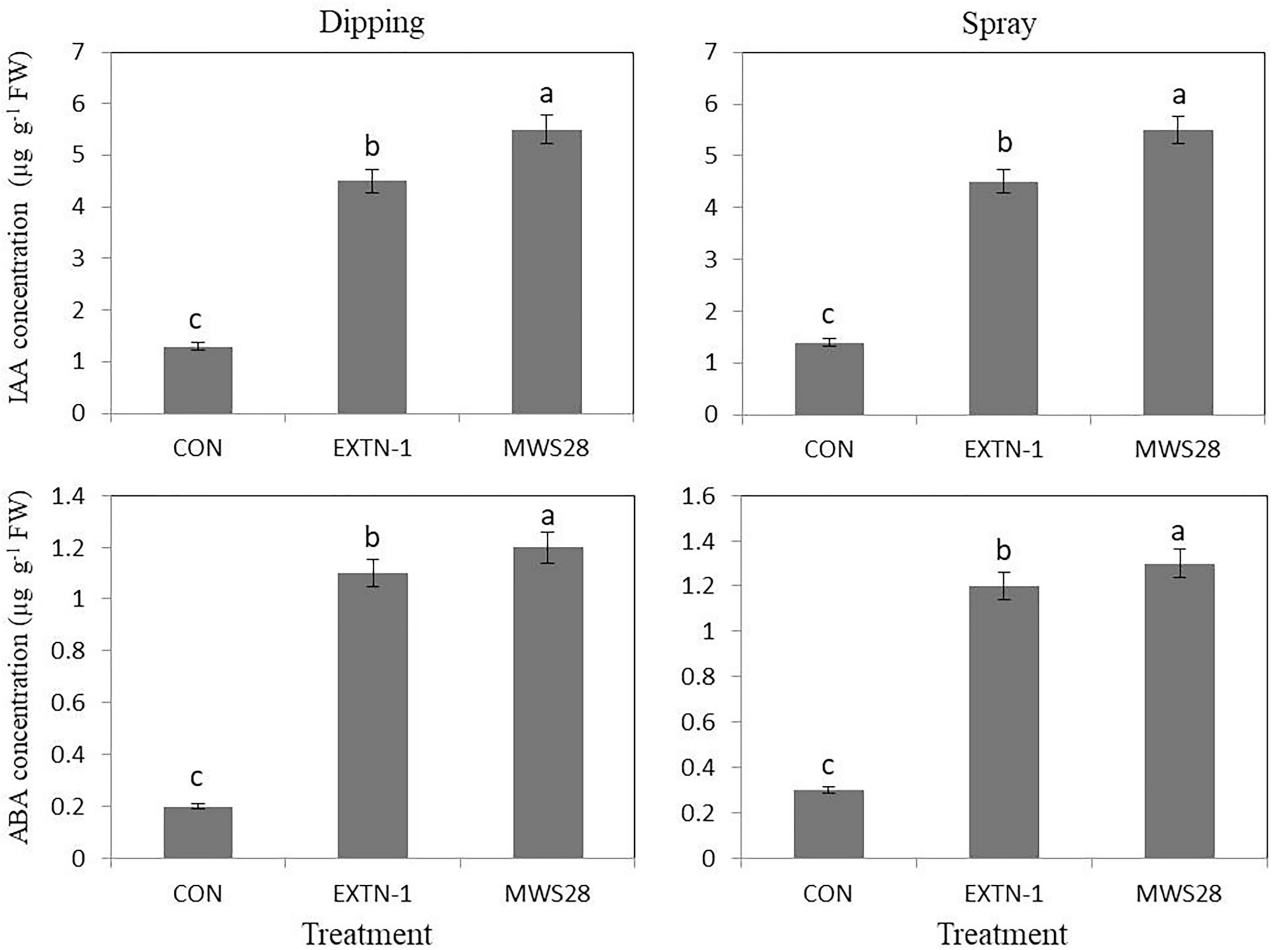
Effect of *Bacillus velezensis* MWS28 application on endogenous indole-3-acetic acid (IAA) and abscisic acid (ABA) contents in apple stem cuttings subjected to chilling stress. Plants were treated with MWS28 bacaterial suspensions through dipping or foliar spray methods and compared with the non-inoculated control (CON) and the positive control strain EXTN-1. IAA and ABA concentrations were determined after chilling treatment and expressed as μg g^−1^ fresh weight (FW). Data represent the mean ± standard error (SE) of five biological replicates (*n* = 5). Different letters above bars indicate significant differences among treatments according to the least significant difference (LSD) test at *P* ≤ 0.05.

Bacterial inoculation significantly altered endogenous phytohormone levels in apple stem cuttings exposed to chilling stress (Figure 6). In both dipping and foliar spray treatments, MWS28-treated plants exhibited the highest indole-3-acetic acid (IAA) concentrations, followed by EXTN-1-treated plants, whereas untreated controls showed the lowest IAA levels. IAA concentrations in MWS28-treated plants were approximately two- to three-fold higher than those observed in the control. In contrast, abscisic acid (ABA) concentrations showed an opposite trend. The highest ABA levels were detected in untreated control plants, while MWS28 treatment resulted in the lowest ABA accumulation in both application methods. EXTN-1 treatment produced intermediate ABA levels between the control and MWS28 treatments. Significant differences among treatments were detected at P ≤ 0.05. These results indicate that MWS28 promotes auxin accumulation while reducing stress-associated ABA accumulation, thereby contributing to improved chilling stress tolerance in apple stem cuttings.

### Effect of MWS28 treatment on stress-related gene expression

**FIGURE 7:**
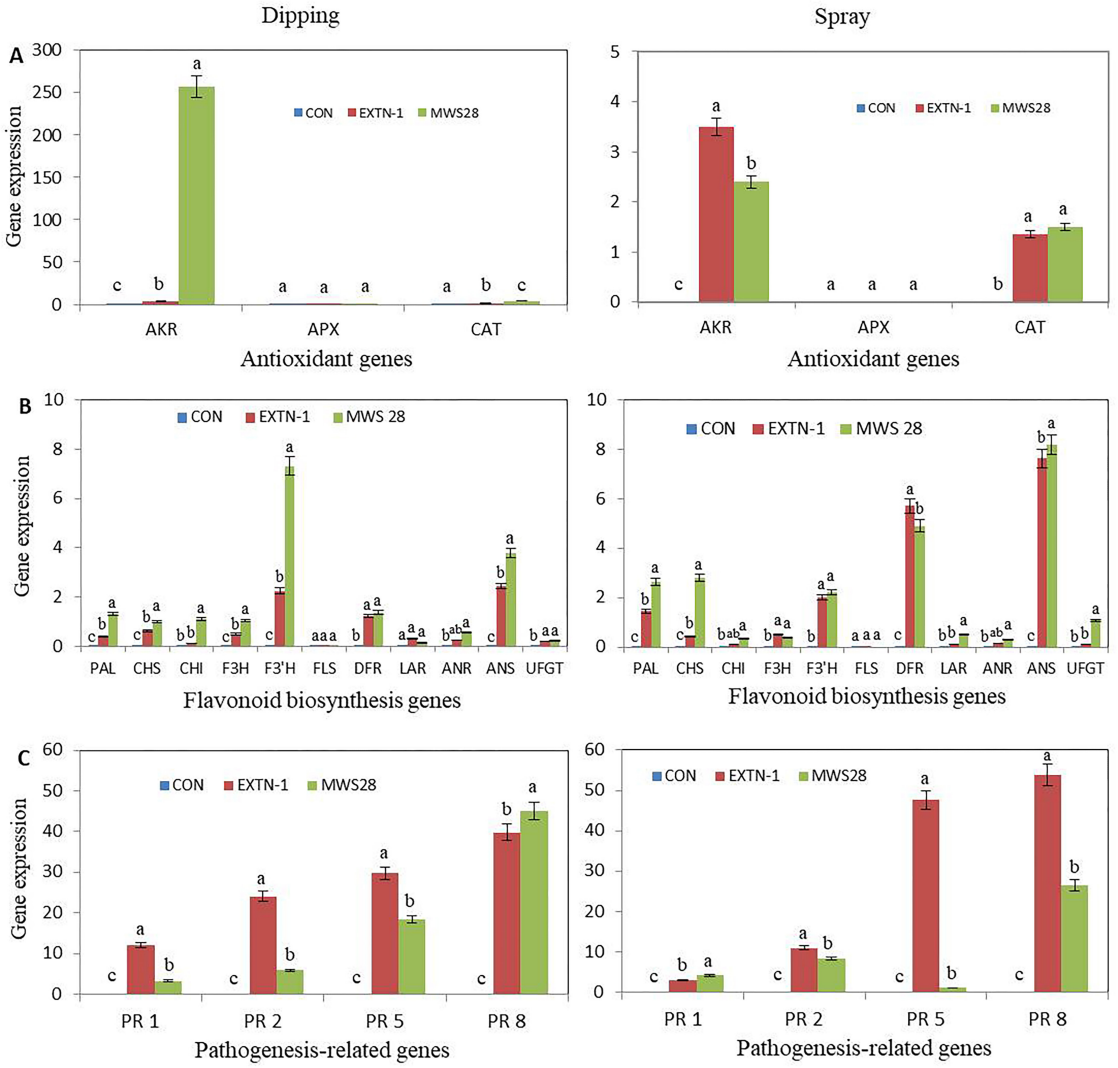
Expression of stress-related genes in “Fuji” apple leaves after bacterial treatments. Relative expression levels of **(A)** antioxidant genes, **(B)** flavonoid biosynthesis genes, and **(C)** pathogenesis-related (PR) genes in “Fuji” apple leaves 24 h post-inoculation (hpi) with *B. velezensis* MWS28 and *B. vallismortis* EXTN-1 (positive control), compared to untreated controls (CON). Treatments were applied using either dipping or spraying methods. Gene expression was quantified by qRT-PCR normalized to the control, and is presented as fold change relative to the untreated control group. Different letters above bars indicate significant differences among treatments according to the least significant difference (LSD) test at *P* ≤ 0.05.

The expression levels of antioxidant, flavonoid biosynthesis, and pathogenesis-related (PR) genes in apple leaves were analyzed 24 h after treatment with *Bacillus velezensis* MWS28 and *B. vallismortis* EXTN-1 (positive control) using either dipping or foliar spray application (Figure 7). Both bacterial treatments significantly (*P* ≤ 0.05) induced the expression of stress-responsive genes compared with untreated control, although the magnitude of induction varied among genes and application methods.

For antioxidant genes, the dipping treatment with MWS28 resulted in a dramatic increase of over 250-fold in AKR gene expression compared to the untreated control (Figure 7A). In contrast, spraying led to only a 3–4-fold increase. The CAT gene showed minimal expression with spray treatments of EXTN-1 and MWS28 but was not expressed with dipping treatments; however, APX gene expression was undetectable in all treatments.

Bacterial treatments also enhanced the expression of genes involved in flavonoid biosynthesis genes (Figure 7B). Across both dipping and foliar spray methods, gene expression levels were consistently higher in MWS28-treated plants compared with EXTN-1-treated plants and untreated controls. Especially, MWS28 treatment via dipping resulted in a 6–7-fold increase in *F3’H* gene expression relative to the untreated control, with *ANS* gene expression showing the next highest increase. In contrast, EXTN-1 treatment showed medium expression of the *F3H*, *DFR* and *ANS* genes. For the spray method, EXTN-1 and MWS28 treatments produced similar expression levels of *F3H*, *DFR*, and *ANS* genes, while no expression of these flavonoid pathway genes was detected in untreated control.

In the case of pathogenesis-related (PR) genes, both MWS28 and EXTN-1 treatments, regardless of applied by dipping or spraying method, induced PR5 and PR8 expression by approximately 50–60-fold compared to the control (Figure 7C). Collectively, these results demonstrate that both MWS28 and EXTN-1 significantly (*P* ≤ 0.05) enhance stress-related gene expression in “Fuji” apple leaves, with the most pronounced effects observed in MWS28-treated plants. The gene expression outcomes varied depending on the method of treatment. These findings provide valuable insights for the development of future agricultural technologies aimed at reducing the impact of climate change.

## Discussion

This study investigated the effects of *Bacillus velezensis* MWS28 and *B. vallismortis* EXTN-1 on the tolerance of apple plants to cold and drought stress. The reduction in cold and chilling injury observed following bacterial treatments are consistent with previous research demonstrating that plant growth-promoting rhizobacteria (PGPR), particularly *Bacillus* spp., enhance plant tolerance to abiotic stresses by improving antioxidant activity and regulating stress-related hormonal pathway (Tsotetsi et al., 2022; Khan et al., 2023). Similar protective effects have been reported for *Bacillus amyloliquefaciens* and related PGPR in various crops exposed to adverse environmental conditions (Chowdhury et al., 2015; Xie et al., 2017). Notably, the strains MWS28 and EXTN-1 in our study provided greater protection against chilling injury than conventional treatments such as BTH or streptomycin, highlighting the potential of microbial-based approaches as sustainable alternatives for abiotic stress management (Vurukonda et al., 2016). These findings collectively indicate that *Bacillus* strains represent promising biological tools for improving cold tolerance in apple and other fruit crops. Although MWS28 and EXTN-1 were originally isolated from rhizosphere soils, their application to apple aerial tissues was based on the well-documented ability of *Bacillus* spp. to colonize aboveground plant tissues, survive under environmental conditions through endospore formation, and induce systemic stress tolerance responses (Lindow and Brandl, 2003; Chowdhury et al., 2015; Hashem et al., 2019). In addition, phyllosphere-applied *Bacillus* strains have been reported to enhance plant tolerance to low-temperature stress and improve physiological resilience under adverse environmental conditions (Han et al., 2025). Therefore, the foliar application strategy used in the present study was supported by previous evidence demonstrating that beneficial *Bacillus* spp. can function effectively beyond the rhizosphere.

The enhanced drought tolerance observed in MWS28- and EXTN-1-treated apple plants is in agreement with previous reports showing that plant PGPR, particularly *Bacillus* species, alleviate drought stress by improving relative moisture content (RMC), osmotic balance, and overall physiological performance (Yang et al., 2009; Etesami et al., 2023). In the present study, bacterial treatments reduced leaf wilting, maintaining higher RMC, and promoted faster recovery following rewatering. Similar responses have been reported in maize, rice, and pepper inoculated with *Bacillus velezensis*, where improved drought resilience was associated with enhanced water retention and stress adaptation (Zhang et al., 2026; Lim and Kim, 2013). The ability of MWS28 and EXTN-1 to sustain higher RMC in apple leaves during drought is consistent with studies showing that *Bacillus*-treated plants display improved physiological and biochemical responses, such as increased production of stress-related phytohormones and antioxidants, which contribute to drought tolerance (Tsotetsi et al., 2022; Vurukonda et al., 2016).

Chilling stress disrupts cellular redox homeostasis, causing excessive reactive oxygen species (ROS) accumulation and oxidative damage in plant tissues. In the present study, inoculation with *Bacillus velezensis* MWS28 significantly increased the activities of APX, CAT, SOD, and POD in apple stem cuttings under chilling stress, indicating an enhanced ROS-scavenging system. Similar improvements in antioxidant defenses have been reported in plants inoculated with plant growth-promoting rhizobacteria (PGPR), particularly *Bacillus* spp., under low-temperature and other abiotic stresses, demonstrating their role in ROS detoxification and stress tolerance (Abd El-Daim et al., 2014; Zhang et al., 2023). The enhanced antioxidant capacity was accompanied by a significant reduction in malondialdehyde (MDA) accumulation, suggesting lower lipid peroxidation and improved membrane stability. Comparable decreases in MDA content have been observed in PGPR-treated plants under abiotic stress due to enhanced antioxidant protection against ROS-induced membrane damage (Guo et al., 2012; Han et al., 2025). Furthermore, MWS28 increased indole-3-acetic acid (IAA) levels while reducing abscisic acid (ABA) accumulation. Such phytohormonal modulation is a common PGPR-mediated mechanism that promotes plant growth and adaptation under stress conditions (Vejan et al., 2016). Collectively, these results indicate that MWS28 enhances chilling tolerance in apple stem cuttings through coordinated activation of antioxidant defenses, reduction of lipid peroxidation, and regulation of endogenous phytohormones, consistent with previous reports on PGPR-mediated cold stress tolerance (Zhang et al., 2023).

The increased expression of antioxidant, flavonoid biosynthesis, and PR genes in “Fuji” apple leaves after treatment with MWS28 and EXTN-1 supports with previous research demonstrating that PGPR improve plant stress tolerance. For example, Etesami et al. (2023) reported that *Bacillus* spp. increased antioxidant gene expression and resistance to abiotic stresses including drought and cold. Such stresses cause an accumulation of reactive oxygen species (ROS), which can damage cellular structures (Tanveer and Ahmed, 2020). In this study, APX and CAT genes exhibited the most pronounced upregulation, especially in response to MWS28 and the positive control, EXTN-1. These genes play key roles in detoxifying hydrogen peroxide (H_2_O_2_), thereby protecting cells from oxidative damage. The marked increase in APX and CAT expression suggests that improved cold and drought tolerance in treated apples is closely linked to improved ROS-scavenging capacity. In contrast, other antioxidant genes like AKR exhibited only moderate changes, indicating a gene-specific response to bacterial treatment.

Zhou W. et al. (2025) demonstrated that *Bacillus* strains enhanced the expression of flavonoid biosynthesis genes, thereby strengthening plant defense. The notable efficacy of the dipping method, particularly with the MWS28 strain, is consistent with findings by Gayithri et al. (2026), where direct application of *Bacillus* strains can increase bacterial colonization and gene activation in host tissues. Flavonoids, synthesized via phenylpropanoid pathway by *PAL*, *CHS*, *CHI*, and *F3H* genes, play important roles in plant adaptation to abiotic stresses such as cold and drought (Rao and Zheng, 2025). Furthermore, PGPR-mediated induction of PR genes is a key component of induced systemic resistance (ISR) Pieterse et al. (2014), supporting the elevated PR gene expression observed in the present study. In apples, low- temperature stress induces PR gene expression, leading to the accumulation of protective proteins that statbilize cell membranes, enhance antioxidant defenses, and strengthen cell wall integrity, thereby mitigating chilling-induced damage (Van Loon et al., 2006; Rejeb et al., 2014).

It is important to note that gene expression and biochemical analyses were conducted at different stages of the experiment. Gene expression was assessed 24 h after bacterial treatment, prior to chilling stress, whereas antioxidant enzyme activities and MDA accumulation were measured after exposure to chilling stress. Therefore, the transcriptional changes observed in this study reflect bacterial-induced priming responses rather than direct responses to cold stress. The pre-stress upregulation of defense- and stress-related genes may have contributed to the enhanced physiological and biochemical performance observed under subsequent chilling conditions. However, direct correlations between gene expression levels and biochemical parameters cannot be established because the measurements were obtained from distinct physiological states and at different time points.

In conclusion, the study demonstrates that bacterial treatments with MWS28 and EXTN-1 (positive control) significantly improved the tolerance of apple plants to both cold and drought stresses. Notably, a discrepancy was observed between physiological and molecular responses depending on the method of bacterial application. Physiological assessments, such as the reduction in chilling injury, indicated that foliar spray was generally more effective. In contrast, molecular analyses revealed that the dipping method, particularly with MWS28, resulted in the strongest gene activation. These findings suggest that foliar spraying may provide immediate protection and reduce visible stress-related symptoms more effectively, while dipping can induce a more robust molecular defense response. These findings highlight the importance of integrating both physiological and molecular evaluations when determining the abiotic stress management strategies in crops. Future research should investigate the mechanisms underlying the stress protective effects, and further studies will also focus on the validation of greenhouse and orchard-scale systems.

## Data Availability

The raw data supporting the conclusions of this article will be made available by the authors, without undue reservation.
